# Pressurized Liquid Extraction of a Phycocyanobilin Chromophore and Its Reconstitution with a Cyanobacteriochrome Photosensor for Efficient Isotopic Labeling

**DOI:** 10.1093/pcp/pcaa164

**Published:** 2021-01-02

**Authors:** Takanari Kamo, Toshihiko Eki, Yuu Hirose

**Affiliations:** Department of Applied Chemistry and Life Science, Toyohashi University of Technology, 1-1 Hibarigaoka, Tempaku, Toyohashi, Aichi, 441-8580 Japan

**Keywords:** Bilin, Isotopic labeling, Linear tetrapyrrole, Phytochrome, Phycocyanin

## Abstract

Linear tetrapyrrole compounds (bilins) are chromophores of the phytochrome and cyanobacteriochrome classes of photosensors and light-harvesting phycobiliproteins. Various spectroscopic techniques, such as resonance Raman, Fourier transform-infrared and nuclear magnetic resonance, have been used to elucidate the structures underlying their remarkable spectral diversity, in which the signals are experimentally assigned to specific structures using isotopically labeled bilin. However, current methods for isotopic labeling of bilins require specialized expertise, time-consuming procedures and/or expensive reagents. To address these shortcomings, we established a method for pressurized liquid extraction of phycocyanobilin (PCB) from the phycobiliprotein powder Lina Blue and also the cyanobacterium *Synechocystis* sp. PCC 6803 (*Synechocystis*). PCB was efficiently cleaved in ethanol with three extractions (5 min each) under nitrogen at 125�C and 100 bars. A prewash at 75�C was effective for removing cellular pigments of *Synechocystis* without PCB cleavage. Liquid chromatography and mass spectrometry suggested that PCB was cleaved in the C3*-E* (majority) and C3*-Z* (partial) configurations. ^15^N- and ^13^C/^15^N-labeled PCBs were prepared from *Synechocystis* cells grown with NaH^13^CO_3_ and/or Na^15^NO_3_, the concentrations of which were optimized based on cell growth and pigmentation. Extracted PCB was reconstituted with a recombinant apoprotein of the cyanobacteriochrome-class photosensor RcaE. Yield of the photoactive holoprotein was improved by optimization of the expression conditions and cell disruption in the presence of Tween 20. Our method can be applied for the isotopic labeling of other PCB-binding proteins and for the commercial production of non-labeled PCB for food, cosmetic and medical applications.

## Introduction

Organisms utilize various kinds of photosensor proteins to regulate numerous physiological responses. Photosynthetic organisms also utilize diverse antenna proteins to convert light energy into chemical energy. The photosensor and antenna proteins harbor chromophores that absorb a specific wavelength(s) of light. Linear tetrapyrrole compounds (bilins) are chromophores utilized by three different protein families: phytochromes, cyanobacteriochromes and phycobiliproteins. Phytochromes are photosensors that typically absorb red and far-red light and are distributed in higher plants as well as fungi, algae and bacteria (Auldridge and Forest 2011, Rockwell and Lagarias 2017b, Rockwell and Lagarias 2020). Cyanobacteriochromes are phytochrome-related photosensors of broad spectral diversity that are widespread among cyanobacteria (Ikeuchi and Ishizuka 2008, Fushimi and Narikawa 2019, Wiltbank and Kehoe 2019, Villafani et al. 2020). Phytochromes utilize biliverdin, phytochromobilin (PΦB) or phycocyanobilin (PCB) as the native chromophore, whereas cyanobacteriochromes utilize PCB or phycoviolobilin (Supplementary Fig. S1). Phytochromes and cyanobacteriochromes photoconvert between two distinct light-absorbing states, which is triggered by the *Z*/*E* photoisomerization at the C15=C16 double bond of the bilin (Rudiger et al. 1983, Narikawa et al. 2013, Song et al. 2014, Lim et al. 2018, Xu et al. 2020). This conformational change in the bilin causes structural changes in the entire protein, leading to light-induced signal transduction. Phycobiliproteins are components of the light-harvesting antenna complex called phycobilisome, which is distributed among cyanobacteria and certain algae, including rhodophytes, cryptophytes and glaucophytes (Watanabe and Ikeuchi 2013, Bryant and Canniffe 2018). PCB is the chromophore of allophycocyanin and phycocyanin, which form the central core and radiating rods, respectively, of phycobilisome and absorb red light. Phycoerythrobilin and phycourobilin are the chromophores of phycoerythrin and form the peripheral components of the rods and extend the light-harvesting capacity of the phycobilisome to the green and blue regions in some species (Supplementary Fig. S1).

The maximal-absorption wavelength of phycobiliproteins is well correlated with the length of the π-conjugated system of bilins as determined by the analysis of crystal structures (Schmidt et al. 2007). On the other hand, phytochromes and cyanobacteriochromes have a relatively broader absorption spectrum, spanning near-UV to far-red light (Rockwell et al. 2014a) (also see the references above). Various factors have been shown to influence the spectral properties of phytochromes and cyanobacteriochromes, including the *Z*/*E* or *syn*/*anti* conformation of the four pyrrole rings (Rudiger et al. 1983, Rockwell et al. 2014b, Xu et al. 2020), protonation/deprotonation of pyrrole nitrogen (Hirose et al. 2013, Sato et al. 2019), stable covalent linkage to the apoprotein (Lamparter et al. 2001), reversible attachment of a second thiol group (Rockwell et al. 2008) and/or the recently proposed di-protonated A-ring lactim (Bandara et al. 2020). Obtaining chemical and structural information for bilins is essential for elucidating the spectral tuning mechanisms of phytochromes and cyanobacteriochromes. For this purpose, vibrational (Raman or Fourier transform-infrared) spectroscopy and various types of nuclear magnetic resonance (NMR) spectroscopies are powerful approaches (Foerstendorf et al. 2000, Mroginski et al. 2011, Lim et al. 2018). Because these measurements yield highly complex spectra, the obtained signals must be experimentally assigned to specific bilin structures based on the isotopic shift using the ^13^C- and/or ^15^N-labeled bilins. The three general approaches for isotopic labeling of bilins are discussed below.

The first approach involves chemical synthesis of bilins (Inomata 2008, Bongards and G�Rtner 2010). In this approach, each of the A-, B, C and D rings of the bilin is synthesized separately, each A–B and C–D block is combined and then the two blocks are combined to form the complete A–B–C–D rings. The B- and C-ring propionates of the bilin are typically converted to the dimethyl ester, but their free-acid forms are also synthesized for PCB (Jayasundera et al. 1998, Kakiuchi et al. 1998) and PΦB (Kakiuchi et al. 1999). This approach guarantees the full labeling with isotope and, in principle, can label all positions of the bilin. Selective ^13^C-labeling was reported at the C10 position of PΦB and at the C5/C10/C15 and C10/C15 positions of PCB (Makhynya et al. 2007). The chemical synthesis approach can also produce non-natural bilin analogs—e.g. biliverdin, PCB and PΦB analogs that are substituted with various side chains (Hanzawa et al. 2001) and biliverdin and PCB analogs that are sterically locked in the configurations/conformations of C5*-Z*,*syn*, C5-*Z*,*anti*, C15-*Z*,*syn*, C15-*E*,*syn*, C15-*Z*,*anti* and/or C15-*E*,*anti* by the formation of dual covalent linkages between the A–B and/or C–D rings (Inomata et al. 2005, Inomata et al. 2006, Nishiyama et al. 2010). However, the chemical synthesis of bilins requires many time-consuming steps and specialized skills, which poses a challenge for most biological laboratories. The synthesized bilin is autocatalytically incorporated into the apoprotein in vitro and yields photoactive holoproteins, but this process can become troubled when the apoprotein has low solubility (Hirose et al. 2013) and/or there are altered spectral properties in the holoprotein owing to protein misfolding (Song et al. 2015).

The second approach is the use of 5-aminolevulinic acid (ALA) with an *Escherichia coli* (*E. coli*) strain that has been genetically engineered to produce bilins. In this approach, ^13^C- and/or ^15^N-labeled ALA is added to the growth medium of *E. coli* cells and is consumed as a precursor for heme biosynthesis. The isotopically labeled heme is converted to biliverdin by the heterologously expressed heme oxygenase (*ho1*) and can be further converted to PCB or PΦB upon expression of the ferredoxin-dependent bilin reductase *pcyA* or *hy2*, respectively (Dammeyer and Frankenberg-Dinkel 2008, Rockwell and Lagarias 2017a). When the target apoprotein is overexpressed with this system, the apoprotein incorporates the labeled bilin and yields a photoactive holoprotein in the *E. coli* cells (Mukougawa et al. 2006). This approach also enables partially selective labeling of carbon atoms of the bilin: the C4/C5/C9/C10/C11/C15/C19 positions are labeled with 5-^13^C-labeled ALA, whereas the C1/C3/C6/C8/C12/C14/C16/C18 positions are labeled with 4-^13^C-labeled ALA (Rockwell et al. 2015). However, isotopically labeled ALA is too expensive for practical use in experiments, e.g. 4-^13^C-labeled ALA (list price: ∼$13,000/g, Cambridge Isotope Laboratories, USA), 5-^13^C-labeled ALA (list price: ∼$19,000/g, Cambridge Isotope Laboratories) and ^13^C- and ^15^N-labeled ALA (list price: ∼$150,000/g, Sigma-Aldrich, USA). In addition, the labeling rate for the isotope in bilin should be validated for some applications to avoid label heterogeneity because of the existence of non-labeled ALA in *E. coli* cells.

The third approach is the extraction of PCB from the phycobiliproteins of phycocyanin-containing cyanobacteria species, e.g. *Synechocystis* sp. PCC 6803 (*Synechocystis*), which are grown in an isotopically labeled medium (Strauss et al. 2005). This is the classic approach, and it can produce PCB that is uniformly labeled with ^13^C and/or ^15^N for relatively low cost using inorganic carbon and nitrogen sources, NaH^13^CO_3_ and Na^15^NO_3_ (each ∼$60/g; Cambridge Isotope Laboratories, Inc.). The extracted PCB has been applied for chemical modification of the B- and C-ring propionates (Shang et al. 2010). However, the PCB extraction approach has limitations. For example, it typically includes a purification process of an intact phycobilisome to eliminate the contamination of other cellular materials, e.g. aqueous two-phase separation followed by sucrose density gradient centrifugation (Kondo et al. 2005), which is time-consuming and produces a lower yield of phycobiliproteins. In addition, the thioether linkage between the PCB and cysteine residues of phycocyanin and allophycocyanin are cleaved with boiling methanol under a nitrogen atmosphere (Chapman et al. 1968), but this process requires ∼16 h for complete reaction (Malwade et al. 2016) and requires toxic HgCl_2_ as the catalyst in some protocols (Terry 2002). Finally, the low yield and/or misfolding of the apoprotein could be problematic in this approach, as was mentioned in the description of the chemical synthesis approach.

Recently, Roda-Serrat et al. (2018) reported that alcohol treatment under high pressure and high temperature efficiently cleaves the PCB from Lina Blue, which is a commercial phycocyanin powder used as a food additive. Lina Blue is boiled in 96% (v/v) ethanol in a sealed vessel for 30 min at 120�C, and both the yield and purity of the extracted PCB are comparable to values obtained with overnight reflux (Roda-Serrat et al. 2018). Inspired by that pioneering study, we developed a method for rapid and efficient extraction of PCB using pressurized liquid extraction (PLE) from Lina Blue and *Synechocystis* cells. We also investigated the effects of ^15^N and ^13^C concentrations on the growth and pigmentation of *Synechocystis*. Finally, we optimized conditions for in vitro reconstitution of PCB prepared by PLE with the cyanobacteriochrome RcaE, whose green and red light sensitivity is indispensable for the regulation of chromatic acclimation in cyanobacteria (Kehoe and Grossman 1996, Hirose et al. 2010, Hirose et al. 2019). Our method is useful for isotopic labeling of diverse PCB-binding proteins and can be applied for the commercial production of non-labeled PCB for the food, cosmetic and medical industries.

## Results

### Effects of temperature and number of extractions on PCB cleavage

PLE, which is also called accelerated solvent extraction, is a technique for extracting target compounds from solid materials into a liquid phase (Mustafa and Turner 2011). We evaluated the effectiveness of PLE for extracting PCB from Lina Blue (Fig.�1). A SpeedExtractor (model E-916; B�CHI Labortechnik AG) was used for PLE (Fig.�1), as it is superior to the sealed-vessel method with strict control of the pressure under a nitrogen atmosphere. The extraction solvent was ethanol, which produces fewer derivatives of PCBs and thus is appropriate for food applications (Roda-Serrat et al. 2018). We optimized the procedure with respect to the number of extractions (1–4 extractions) and temperature (100, 125, 150 or 200�C) with 5-min extractions at 100 bars. The amount of extracted PCB was estimated by absorption at 660 nm. For all investigated temperatures, the amount of extracted PCB was greatest in the 1st extract and gradually decreased for the 2nd, 3rd and 4th extractions (Fig.�2B). This observation is consistent with that the cleavage of PCB showed peudo-first-order kinetics (Malwade et al. 2016). For the 1st extraction, PCB yield at 100�C was lower than that at 125, 150 and 200�C, although the yield for each of the 3rd and 4th extractions at 100�C was higher than that at 125, 150 or 200�C (Fig.�2B), suggesting that PLE at 100�C is not effective for cleaving the covalent linkage of PCB. The PCB yields for the 1st extraction at 125 and 150�C were ∼3-fold higher than those at 100�C (Fig.�2B). The PCB yields for the 1st through 4th extractions were highest at 125�C (Fig.�2C), indicating that this is the optimum temperature for PCB extraction. The PCB yields for the 1st through 4th extractions were lower at 200�C than at 125 and 150�C (Fig.�2B, C), suggesting that PLE at 200�C resulted in PCB degradation.

**Fig. 1:**
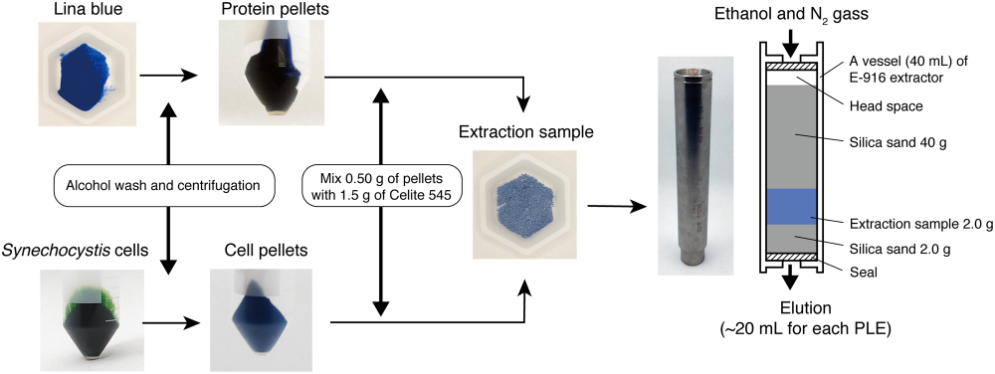
Flowchart of PLE of PCB from Lina Blue (left upper) or *Synechocystis* (left lower). In both cases, alcohol-washed samples are mixed with Celite and subjected to PLE in a vessel on the E-916 speed extractor (right photograph). In each cycle, the vessel is filled with ethanol (∼20 ml) and heated at set temperature for 5 min under nitrogen and 100 bars and eluted with a flush of nitrogen gas.

**Fig. 2:**
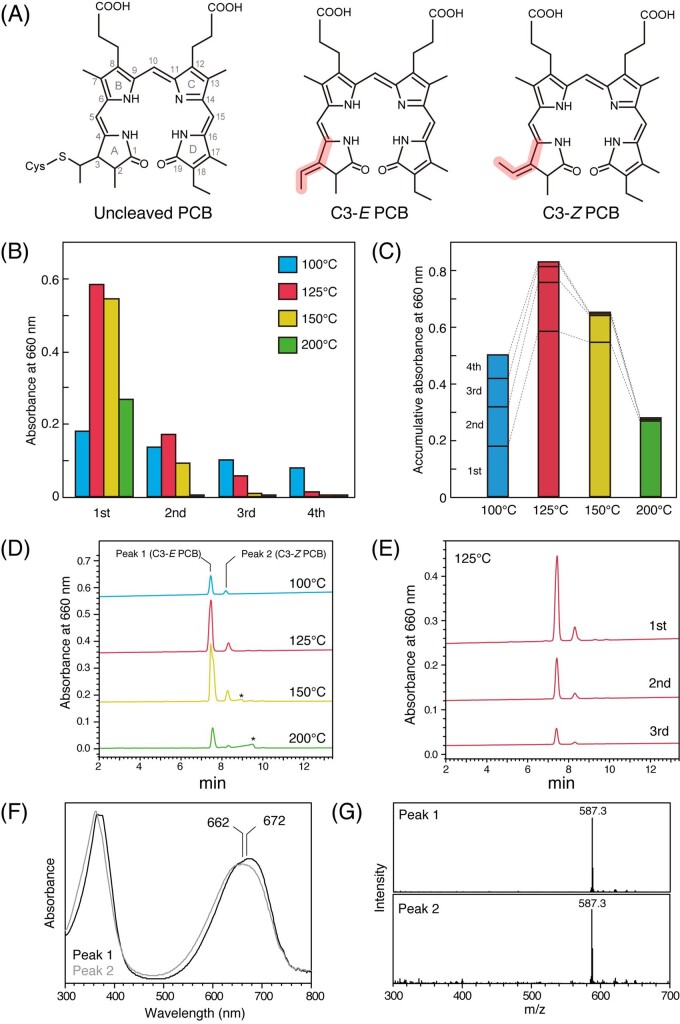
PLE of PCB from Lina Blue. (A) Structures of PCB covalently linked to denatured phycobiliproteins (left), C3*-E* PCB (center) and C3*-Z* PCB (right). The C3*-E* and C3*-Z* structures are shown in red. (B) Absorbance at 660 nm of PCB isolated from the 1st, 2nd, 3rd and 4th extractions at 100�C (blue), 125�C (red), 150�C (yellow) and 200�C (green). (C) Cumulative values of the absorbance at 660 nm in the 1st, 2nd, 3rd and 4th extractions are shown for each temperature. (D) HPLC chromatogram of the 1st extraction at 100, 125, 150 or 200�C. The peaks of the putative degraded PCB are indicated with asterisks. (E) HPLC chromatogram of the 1st, 2nd and 3rd extractions at 125�C. (F) Absorption spectra for peak 1 and peak 2 in the chromatogram for the 1st extraction at 125�C. (G) MS spectra for peak 1 (upper) and peak 2 (lower) in the chromatogram for the 1st extraction at 125�C.

We used liquid chromatography–mass spectrometry (LC–MS) to quantify PCB in each round of extraction. We observed two major peaks under all extraction conditions; peak 1 eluted at 7.6 min and peak 2 eluted at 8.4 min (Fig.�2D). The ratio of the absorbance of peak 1 to peak 2 was 7.5:1 for the 1st extraction at 125�C, and this ratio remained almost constant at each temperature or all rounds of extraction (Fig.�2D, E). The absorption maximum of peak 1 was 672 nm, whereas that of peak 2 was slightly blue-shifted at 662 nm (Fig.�2F). Both peaks corresponded to the same molecular weight, *m*/*z* 587.3, as determined with electron-ionization MS (Fig.�2G), which corresponds to a quasi-molecular ion of PCB, *m*/*z* 587 [M+H]^+^. These results were consistent with the HPLC chromatogram and NMR assignment reported previously, and thus, peak 1 could be assigned as C3*-E* PCB and peak 2 as C3*-Z* PCB (Roda-Serrat et al. 2018). Extractions carried out at 150 and 200�C showed minor peaks (Fig.�2D, asterisk), which may represent degraded PCBs. The PCB extract showed a red-absorption peak at 694 nm in 8 M urea, pH 2.0, which was red-shifted from the denatured Lina Blue by 31 nm in the same solvent (Supplementary Fig. S2). This red shift is the signature of ethylidene group formation at the A ring upon cleavage of PCB (Fig.�2A). The efficiency of PCB cleavage was calculated as 53 � 3.3% (mean � standard deviation; *n* = 3) with PLE at 125�C for three extractions, which is based on the heuristic assumption that the mixtures of uncleaved PCB and the extracted C3*-E* and C3*-Z* PCB have the same molecular extinction coefficient in their red-absorption peaks in 8 M urea pH 2.0. The residual PCB after four PLEs at 125�C had a main absorption peak at 666 nm in 8 M urea pH 2.0 (Supplementary Fig. S2), indicating the presence of covalently linked PCB that was not accessible to solvent during PLE.

### Effect of carbon and nitrogen concentrations on the phycocyanin content

The ^15^N- and ^13^C/^15^N-labeled PCBs were produced in the unicellular cyanobacterium *Synechocystis*. The phycobilisome of *Synechocystis* consists of phycocyanin rods and an allophycocyanin core, which covalently bind PCB as a chromophore (Scheer and Zhao 2008). For ^15^N labeling, *Synechocystis* cells were grown in BG11 medium containing Na^15^NO_3_ with CO_2_ aeration. For the ^13^C/^15^N labeling, the cells were grown in a sealed bottle with modified BG11 medium containing NaH^13^CO_3_, Na^15^NO_3_ and N-cyclohexyl-2-aminoethanesulfonic acid (CHES) buffer that mitigates the increase of pH caused by the consumption of ${\text{HCO}}_{3}^{-}$ (Strauss et al. 2005). To optimize the PCB yield under the atypical growth conditions for ^13^C/^15^N-labeling, we investigated the effects of the concentrations of NaHCO_3_ and NaNO_3_ on cell density and content of each of chlorophyll *a* and phycocyanin by absorption spectroscopy. A total of 16 conditions with different concentrations of NaHCO_3_ and NaNO_3_ were investigated: 100, 50, 25 and 12.5 mM for NaHCO_3_ and 17.4 (nearly equal to the original concentration of BG11), 8.72, 4.36 and 2.18 mM for NaNO_3_ (Fig.�3A). In subsequent text, we refer to the concentrations of NaHCO_3_ and NaNO_3_ as mM^C^ and mM^N^, respectively.

**Fig. 3:**
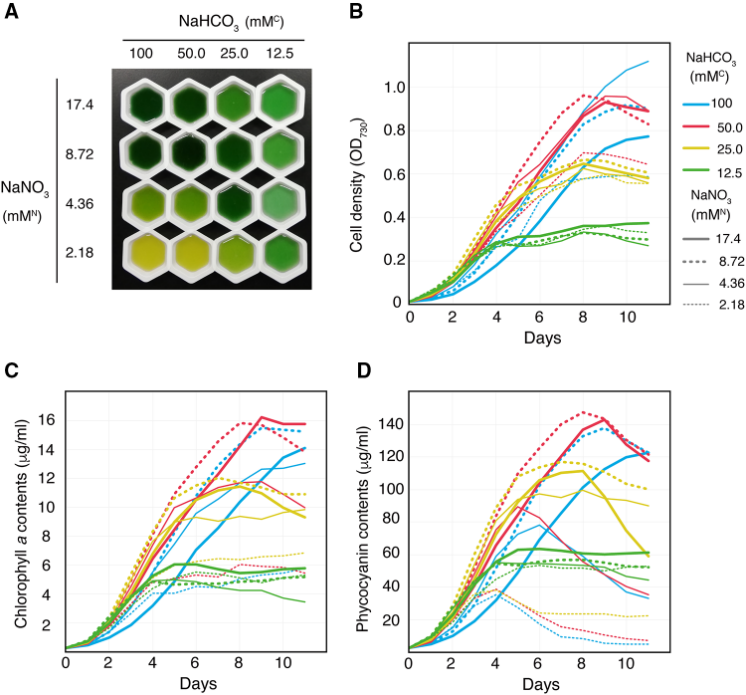
Effects of carbon and nitrogen concentrations on the growth and pigmentation of *Synechocystis*. (A) Photograph of *Synechocystis* cell cultures harvested on day 11. Concentrations of NaHCO_3_ and NaNO_3_ are shown. Cell density (B), chlorophyll *a* (C) and phycocyanin (D) content, as estimated by absorption spectroscopy. The OD_730_ value in (B) was measured with an integrating sphere and gave lower values of OD_730_ compared with those measured with a conventional UV–Vis spectrometer. (B–D) The different concentrations of NaHCO_3_ and NaNO_3_ are shown as colored lines according to the legend.

Cell densities monitored at OD_730_ plateaued within 11 d under most conditions. Cell densities under the conditions of 12.5 mM^C^ and 25 mM^C^ almost plateaued at OD_730_ ∼0.3 and ∼0.6, respectively (Fig.�3B, green and yellow lines), which suggests that the carbon concentration limited cell growth under these conditions. On the other hand, cell densities for 50–100 mM^C^ and 2.18 mM^N^ plateaued at OD_730_ 0.6–0.7, which was lower than the densities for the same carbon concentration and 4.42–17.4 mM^N^ (Fig.�3B, red lines). This suggests that the nitrogen concentration limited cell growth under these conditions. The maximum cell density was observed with 50 mM^C^ and 4.42–17.4 mM^N^ and 100 mM^C^ and 4.42–8.72 mM^N^ (Fig.�3B). Compared with these conditions, cell densities for 100 mM^C^ and 17.4 mM^N^ indicated a decrease in growth from the beginning of the cultivation, suggesting that high concentrations of NaHCO_3_ and NaNO_3_ decrease the growth rate of *Synechocystis* (Fig.�3B, thick cyan line). The chlorophyll *a* content was substantially lower for 25–50 mM^C^ and 2.18 mM^N^ than that for the same carbon concentration range and 4.42–17.4 mM^N^ (Fig.�3C). Chlorophyll *a* content was slightly lower for 25–100 mM^C^ and 4.42 mM^N^ than for the same carbon concentration range and 8.72 mM^N^ (Fig.�3C), suggesting that the low-nitrogen content moderately decreased the chlorophyll *a* content. Changes in the chlorophyll *a* content of other samples were consistent with changes in cell density (Fig.�3B, C).

The phycocyanin content, which is the most important factor for maximizing PCB yield, was severely affected by the concentrations of the carbon and nitrogen sources and the cultivation period (Fig.�3D). The phycocyanin content for 25–100 mM^C^ and 2.18 mM^N^ increased until day 4 and then gradually decreased through day 7 (Fig.�3D, thin dashed lines), and the decrease was more pronounced as the carbon concentration increased. Conditions of 50–100 mM^C^ and 4.42 mM^N^ resulted in a similar transition, with the maximum phycocyanin content on days 5–6. Cell cultures under these low-nitrogen conditions were colored green to yellow owing to the low content of phycocyanin (Fig.�3A, Supplementary Fig. S3), which is called chlorosis (Allen and Smith 1969) and is the typical response of cyanobacteria under prolonged nitrogen starvation via phycobilisome degradation for use as a nitrogen source (Giner-Lamia et al. 2017). However, the phycocyanin content also decreased with 25 mM^C^ and 17.4 mM^N^ on days 8–11 (Fig.�3D), for which the cell density growth was limited by the carbon concentration but not by the nitrogen concentration (Fig.�3B). This suggested that carbon starvation caused phycobilisome degradation even when nitrogen was not limited. Taken together, these data suggested that there are suitable ranges for each of carbon and nitrogen concentration for optimizing PCB production. The similarity in prices of NaH^13^CO_3_ and Na^15^NO_3_ (list price for each, ∼$60/g; Cambridge Isotope Laboratories) and the high C:N ratio of the modified BG11 medium indicated that carbon concentration is the primary factor to consider for cost-effective isotopic labeling of PCB. Phycocyanin content increased almost proportionally to the NaHCO_3_ concentration for 25 mM^C^ and 4.42–8.82 mM^N^ and for 12. 5 mM^C^ and 2.21–17.4 mM^N^ (Fig.�3D). Here, we chose 12.5 mM^C^ and 2.21 mM^N^ for the production of 13C/15N-labeled PCB, as the phycocyanin content was stable for long periods (Fig.�3D, thin dashed green line).

### PCB extraction from cyanobacteria cells

PCB extraction from cyanobacteria cells typically includes additional steps (e.g. aqueous two-phase separation, sucrose density gradient ultracentrifugation and/or salt precipitation) to purify the phycobiliproteins. As these steps are time-consuming and decrease the PCB yield, we omitted these steps and extracted the PCB directly from *Synechocystis* cells. *Synechocystis* cells were harvested by centrifugation, washed with ethanol and methanol and subjected to PLE (Fig.�1). We included a prewash extraction at 75�C before the three extraction steps at 125�C. The prewash yielded a yellow-colored solution even an after extensive washing with methanol/ethanol at room temperature before PLE (Figs.�1, 4A). The absorption spectra revealed that the yellow-colored fraction mainly contained carotenoids that had peak absorbance at 452 and 475 nm (Fig.�4B). LC with gradient elution over a relatively higher acetonitrile concentration revealed that this fraction also contained protoporphyrin IX and degraded bilins (Supplementary Fig. S4). The amounts of C3*-E* PCB and C3*-Z* PCB in the prewash fraction were negligible compared with the amounts measured at 125�C (Fig.�4C), indicating that the prewash step could remove the insoluble materials that were not extracted at room temperature under atmospheric pressure. After the prewash, the cell material was subjected to three PLEs at 125�C, as shown in the extraction from Lina Blue (Fig.�2C). The PCB extracts prepared from cyanobacterial cells had the same elution profile obtained for Lina Blue (Figs.�2C, 4C), suggesting that PCB was cleaved mostly in the C3*-E* and partially in C3*-Z* configurations. No other substantive contamination was observed in the LC chromatogram of PCB after PLE at 125�C (Fig.�4B, C).

**Fig. 4:**
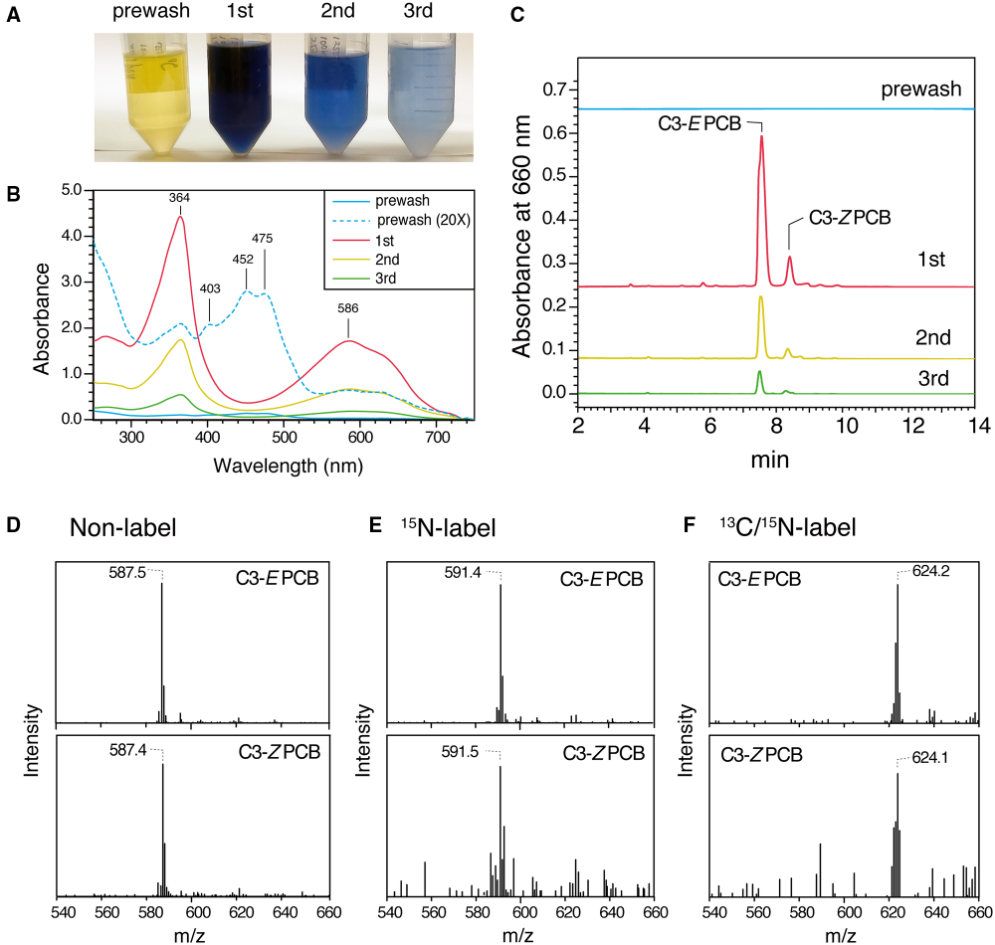
Extraction of non-labeled and ^15^N- and ^15^N/^13^C-labeled PCB from *Synechocystis* cells. Photographs (A) and absorption spectra (B) of PCB extracts for each PLE. (C) HPLC chromatograms of absorbance at 660 nm are shown for the prewash at 75�C and the 1st, 2nd and 3rd extractions from non-labeled cells at 125�C. MS of C3*-E* PCB (upper) and C3*-Z* PCB (lower) prepared from non-labeled (D), ^**1**^^5^N-labeled (E) and ^15^N/^13^C-labeled (F) cells.

Using PLE, we extracted isotopically labeled PCB from the *Synechocysti*s cells grown in modified BG11 medium containing 4.42 mM^N^ for ^15^N labeling or 12.5 mM^C^/2.21 mM^N^ for ^13^ C/^15^N labeling. The LC chromatograms of PCB extracted from ^15^N- and ^13^ C/^15^N-labeled cells showed the same pattern as that from non-labeled cells (Supplementary Fig. S5A, B). The absorption spectra for C3-*E* PCB and C3-*Z* PCB were almost identical to those obtained for non-labeled cells when the experimental noise was subtracted (Supplementary Fig. S5C, D). The *m*/*z* 591.4 of C3-*E* PCB extracted from ^15^N-labeled cells was larger (by 4.1) than the *m*/*z* 587.5 of C3-*E* PCB extracted from non-labeled cells (Fig.�4B, C), indicating that four nitrogen atoms of *3E-*PCB were fully labeled with ^15^N. The *m*/*z* 624.2 of C3-*E* PCB extracted from ^13^ C/^15^N-labeled cells was larger (by 36.7) than the *m*/*z* 587.5 of C3-*E* PCB extracted from non-labeled cells (Fig.�4B, D), indicating that the 4 nitrogen atoms and 33 carbon atoms of PCB were fully labeled with ^15^N and ^13^C, respectively. The same increases in the *m*/*z* values were observed for C3-*Z* PCB extracted from ^15^N-labeled cells and ^13^ C/^15^N-labeled cells (Fig.�4D–F), although they had higher background noise in the MS analysis owing to their low concentrations. Thus, we established an efficient and rapid method for extracting isotopically labeled PCB from *Synechocystis* cells using PLE.

### In vitro reconstitution of PCB with the apoprotein of RcaE

The PCB extracted from Lina Blue was used for in vitro reconstitution with the cyanobacteriochrome RcaE, which undergoes a reversible photoconversion between a green-absorbing state (Pg) and a red-absorbing state (Pr) (Hirose et al. 2008, Hirose et al. 2013). We previously demonstrated that the green/red photoconversion of RcaE is caused by C15*-Z*/C15*-E* photoisomerization and subsequent protonation/deprotonation in PCB, and we named this a protochromic photocycle because the absorption peak maxima are exclusively determined by the protonation state of PCB (Hirose et al. 2013). The details of the photoconversion mechanism of RcaE have been analyzed using time-resolved spectroscopy (Chang et al. 2016, Gottlieb et al. 2013) and resonance Raman spectroscopy (Osoegawa 2019). We also reported the in vitro reconstitution of the RcaE apoprotein with PCB prepared by conventional methanolysis or with a synthetic PCB analog sterically locked in C15*-Z*,*anti* (Nishiyama et al. 2010, Hirose et al. 2013). We found that the RcaE apoprotein was mostly expressed in an insoluble fraction, and only small amounts of the holoprotein could be reconstituted with PCB and the C15*-Z*,*anti* PCB analog, the yield of which was insufficient for resonance Raman or NMR analysis.

To improve the yield of the holoprotein during the in vitro reconstitution of RcaE, we optimized the expression conditions for the RcaE apoprotein in *E. coli* cells. The RcaE apoprotein was overexpressed in *E. coli* cells with different IPTG concentrations (0.01 or 1 mM) at different temperatures (16, 25 or 37�C). The *E. coli* were then disrupted in the presence of 1 mM DTT and 0.1% (v/v) Tween 20, and PCB (prepared by PLE) was added. The photoactive holoprotein was quantified by taking the difference in the absorption spectra of the supernatant of the *E. coli* lysate after illumination with green or red light (Supplementary Fig. S4). The highest yield of holoprotein was obtained with expression at 16�C with 0.01 mM IPTG (Fig.�5A, Supplementary Fig. S7A). We also estimated the effects of the concentrations of DTT, Tween 20 and Triton X-100, which were added after cell disruption, on the yield of holoprotein formation. The addition of 1 or 10 mM of DTT only slightly increased the holoprotein yield (Fig.�5B), suggesting that holoprotein formation is not limited by the oxidation of cysteine residues for covalent linkage to PCB or by disulfide bond formation in the apoprotein. The addition of 0.1 or 1% (v/v) of Tween 20 increased the holoprotein yield by ∼1.4-fold (Fig.�5B). The addition of 0.1 or 1% (v/v) of Triton X-100 enhanced protoporphyrin IX formation but did not substantially improve the holoprotein yield (Supplementary Fig. S6B) (Fischer et al. 2005). Interestingly, we noticed that the timing of the addition of DTT and Tween 20 was critical for achieving a high yield of holoprotein. The difference in the absorption spectra of the Pr peak was ∼0.08 when the *E. coli* were disrupted with French press in the presence of DTT and Tween 20 (Fig.�5A), whereas this value decreased to 0.04–0.05 with the addition of these reagents after cell disruption (Fig.�5B, C). The difference was reproducible and suggested that the use of Tween 20 under high pressure increases the proportion of apoproteins in the soluble fraction, and thus, the apoproteins are capable of incorporating PCB and the holoprotein yield increases. The holoprotein yield increased with increasing concentration of added PCB and plateaued at 1.6 �M of PCB (Fig.�5C). The time course of the covalent linkage of PCB, which was estimated based on the fluorescence intensity of the SDS-PAGE gel, revealed that covalent linkage formation was rapid and completes almost within 2 min (Fig.�5D).

**Fig. 5:**
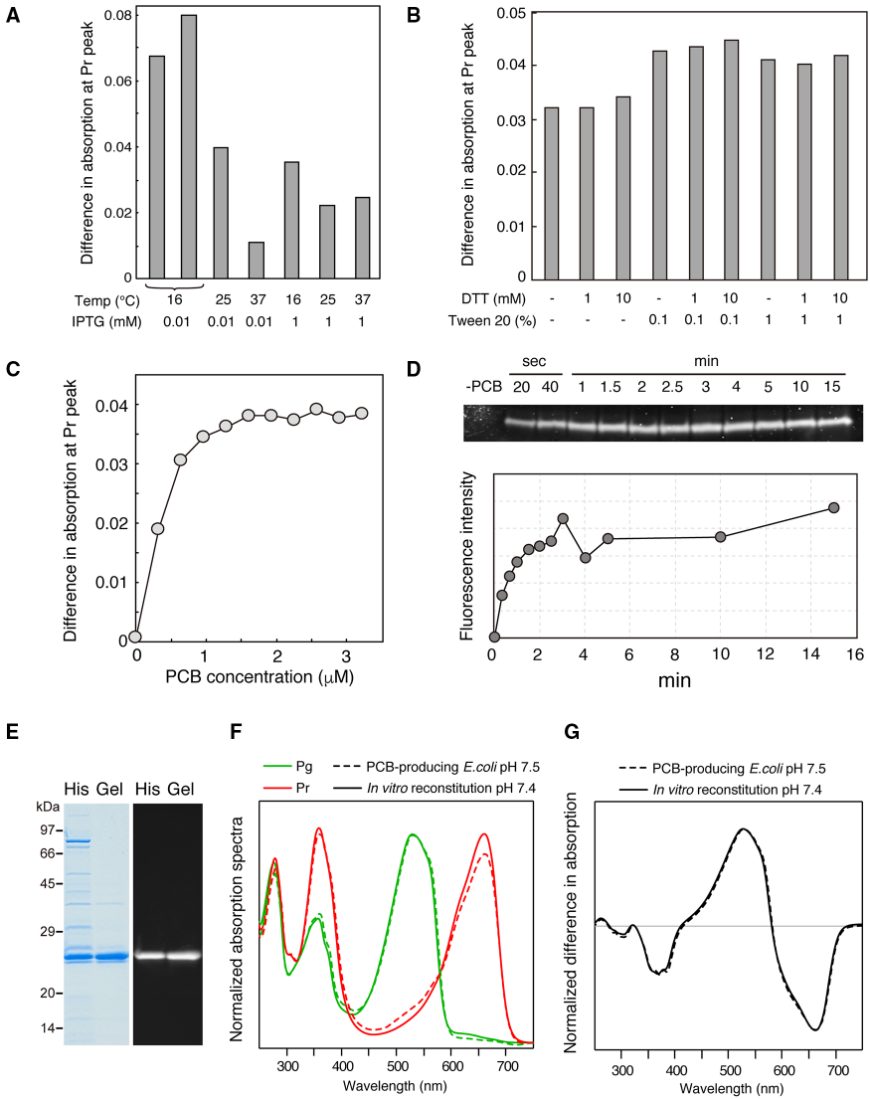
In vitro reconstitution of the extracted PCB with the apoprotein of RcaE. (A and B) The amount of the holoprotein of RcaE in the *E. coli* lysate was estimated by the difference in the absorption maximum of Pr peak (A_660_) after illumination with green or red light. The RcaE apoprotein was expressed at different temperatures and IPTG concentrations (A) or in the presence of different DTT and Tween 20 concentrations (B) as shown. (C) Plots for the difference in the absorption maximum of the Pr peak for different amounts of PCB. (D) Time course of the formation of a covalent linkage of PCB was monitored as the fluorescence of PCB in the acrylamide gel after SDS-PAGE (upper). The intensity of each band was quantified and plotted (lower). (E) Protein purity of the holoproteins after Ni-affinity chromatography (His) and subsequent gel-filtration chromatography (Gel) are shown with CBB staining (left) and fluorescence (right) of the acrylamide gel after SDS-PAGE. (F) Absorption spectra of the Pg (green line) and Pr (red line) of the RcaE holoprotein prepared from the in vitro reconstitution (solid line) and PCB-producing *E. coli* (dashed line). (G) Different absorption spectra of Pg minus Pr prepared from the in vitro reconstitution (solid line) and PCB-producing *E. coli* (dashed line). Spectra were normalized at the Pg peak. Different pH values of the HEPES buffer in the preparation of the holoproteins are shown.

From these results, we concluded that the optimum conditions for the in vitro reconstitution of RcaE consist of the expression of the apoprotein at 16�C with 0.01 mM IPTG, the disruption of *E. coli* cells in the presence of 1 mM DTT and 0.1% (v/v) Tween 20 and the reconstitution of the apoprotein with excess PCB in the *E. coli* lysate with an incubation period of at least 10 min on ice. The holoprotein reconstituted under these conditions was purified with Ni-affinity chromatography and further purified with gel-filtration chromatography. The protein purity and covalent linkage of PCB at each purification step were confirmed by Coomassie brilliant blue (CBB) staining and Zn-enhanced fluorescence of the acrylamide gel after SDS-PAGE (Fig.�5E). The absorption spectra for Pg and Pr of the in vitro-reconstituted holoprotein and their relative intensity compared with the 280 nm peak were comparable with those prepared from PCB-producing *E. coli* in our previous study (Fig.�5F) (Hirose et al. 2013). A slight increase in the Pr peak and the slight decrease of the Pg peak were observed at pH 7.4 (present study) compared with pH 7.5 (previous study) (Fig.�5F) (Hirose et al. 2013). The difference is attributable to the pH sensitivity of the RcaE holoprotein: the photoactive Pr harboring protonated *15E* PCB is in equilibrium with the photoinactive Pg harboring deprotonated C15-*E* PCB at the two pKa values of 7.9 and 8.9 (Hirose et al. 2013). The difference absorption spectrum, i.e. Pg–Pr, was indistinguishable between the holoproteins prepared via in vitro reconstitution and the PCB-producing *E. coli* (Fig.�5G), suggesting that the two preparations yielded photochemically equivalent holoproteins. Finally, the holoprotein yield was calculated as 1.7 mg from 1 l Luria–Bertani medium, and scale up was possible so that a sufficient amount of the holoprotein could be used for Raman and NMR studies, which require a high concentration of protein.

## Discussion

In this study, we established a rapid and efficient method for PCB extraction from phycobiliproteins of cyanobacteria. In our method, PCB yield was 53.3% in Lina Blue, with the heuristic assumption that free and covalently linked PCBs have the same molecular extinction coefficient at their Pr peaks under 8 M urea pH 2.0 (Supplementary Fig. S2). Crespi et al. (1968) reported a yield of 40–50% for PCB cleavage using conventional methanolysis with the assumption that PCB constitutes 4.0% (w/w) of the total phycocyanin. Roda-Serrat et al. (2018) reported that the yield of PCB cleavage was 20 mg/g phycocyanin using PLE with a sealed vessel for 30 min at 120�C and reported a similar value for overnight methanolysis. The value of 20 mg/g phycocyanin corresponds to a 50% yield with the assumption that the chromophore constitutes 4.0% (w/w) of the total phycocyanin (Crespi et al. 1968). Therefore, we assume that the yield of PCB via PLE in our method is comparable to that of conventional methanolysis and PLE using the sealed vessel. The absorption peak of the acid-denatured residual PCB suggests that the residual PCB was attached to cysteine residues of phycobiliproteins (Supplementary Fig. S2). One possible explanation is that denatured phycobiliproteins in the alcohol formed insoluble aggregates, which prevents the solvent from accessing the protein matrix. Additional treatment of the native phycobiliprotein, e.g. by sonication or proteases, before PLE and/or optimization of the solvent composition will be required to further increase the yield of extracted PCB.

PLE of PCB may contribute to the commercial production of free PCB using cyanobacteria cells as the starting material. We used ethanol as solvent because it has been approved for food applications and can be recycled from each extract by distillation. Phycocyanin has been used as a food additive as one of the few natural blue colorants (Newsome et al. 2014). Because phycocyanin is a protein, it is less stable than synthetic colorants with respect to exposure to light, high temperature and acidic conditions (Jespersen et al. 2005). By contrast, the PLE-produced, cleaved PCB is free from the apoprotein matrix and thus is more robust to heat and acid (Fig.�2, Supplementary Fig. S2) and has the potential to complement the limitations of phycocyanin. Because the stability of the absorption spectra of free PCB changes depending on the conditions, e.g. solvent, temperature, pH, and atmosphere, further optimization will be required to develop a free PCB-based food colorant. PCB has antioxidant, anticancer, anti-inflammatory activities (reviewed in Pagels et al. 2019). Based on studies with rodents, McCarty (2007) proposed that daily ingestion of two heaping tablespoons (∼30 g) of *Spirulina* powder would be required to achieve substantive antioxidant activity in humans. The 30 g of *Spirulina* powder may contain up to 5.3 g phycocyanin and 0.21 g PCB based on values of 17.5% (w/w) for phycocyanin content in the cell powder (Patel et al. 2005) and 4.0% (w/w) for PCB content in phycocyanin (Crespi et al. 1968). Therefore, the use of free PCB would enable a 143- and 25-fold enrichment of PCB concentration (w/w) versus *Spirulina* powder and purified phycocyanin, respectively, and could facilitate consumption by humans.

Purified PCB can be used for isotopic labeling of bilin-binding proteins. For this purpose, we investigated the effects of concentration of each of the nitrogen and carbon sources on the phycocyanin content of *Synechocystis*. Phycocyanin content decreased under the high-carbon and low-nitrogen conditions of 25–100 mM^C^ and 2.21 mM^N^ and of 50–100 mM^C^ and 4.42 mM^N^ (Fig.�3D), for which the chlorophyll content and cell density almost plateaued (Fig.�3B, C). The C:N ratio of these conditions ranged from 11.3 to 45.2, which is much higher than the 4.0–4.5 range for the C:N ratio of *Synechocystis* cells (Ch�vez et al. 1999). These points suggest that (i) a decrease in phycocyanin content under these conditions is caused by nitrogen starvation and (ii) phycocyanin rather than chlorophylls is preferentially degraded under nitrogen starvation. In *Anacystis nidulans*, the degradation of phycobilisome occurs after 20 h with the transition to nitrogen starvation, whereas chlorophyll degrades after 40 h with the transition to nitrogen starvation (Allen and Smith 1969). In *Synechocystis*, each of the phycocyanin and chlorophyll content decreased to ∼10% and ∼60%, respectively, after 72 h with the transition to nitrogen starvation (Li and Sherman 2002). Our observation is consistent with these previous reports. On the other hand, a decrease in phycocyanin content was observed under the condition of 25 mM^C^ and 17.4 mM^N^ (Fig.�3D) in concert with a decrease in chlorophyll content and cell density (Fig.�3C). The 1.4 C:N ratio of this condition is much lower than the C:N ratio of *Synechocystis* cells (Ch�vez et al. 1999). Therefore, a decrease in phycocyanin content under this condition was likely caused by carbon starvation. In the thermophilic cyanobacterium *Synechococcus lividus* OH-53s (equivalent to *Synechococcus* sp. PCC 6716), the content of both phycocyanin and chlorophylls decreased after 96 h with the depletion of CO_2_ (Miller and Holt 1977) that accompanies the loss of the thylakoid membrane. These results are consistent with those of our current study. In cyanobacteria, 2-oxoglutarate, which is an intermediate from the Krebs cycle, serves as a signal of nitrogen limitation, whereas 2-phosphoglycolate, which is an intermediate from photorespiration, acts as a signal for inorganic carbon limitation (Zhang et al. 2018). These molecules probably maintain the synthesis and degradation of phycocyanin in *Synechocystis*.

We optimized the conditions for the in vitro reconstitution of PCB prepared by PLE using the green/red cyanobacteriochrome RcaE as an example. In general, holoprotein formation of the phytochrome and cyanobacteriochromes consists of two steps: incorporation of the bilin and subsequent formation of the stable thioether linkage between a cysteine residue and the bilin (Li et al. 1995, Lamparter et al. 2001). In the in vitro reconstitution of phytochrome Cph1, which utilized purified apoprotein, non-covalently bound PCB was detected as the absorption shoulder at 694 nm, which was red-shifted by ∼30 nm from the covalently linked PCB at 665 nm (Lamparter et al. 2001). We reconstituted RcaE with PCB in *E. coli* lysates and did not detect such a red shift by spectroscopy. Purification of the apoprotein is required before addition of PCB to detect the intermediate state of RcaE. In addition, it might be required to treat the RcaE apoprotein with a reagent that blocks cysteine residues, such as iodoacetamide, to trap such an intermediate because the covalent linkage formation of PCB is a rapid reaction that reaches completion within 2 min (Fig.�5D).

We observed that the optimization of growth temperature and IPTG concentration greatly affected the yield of the RcaE holoprotein, in that there was an 8-fold difference between 16 and 37�C, with IPTG induction (0.01 mM, Fig.�5A). Disruption of *E. coli* cells with French press in the presence of Tween 20 contributed to an approximate 2-fold improvement in yield (Fig.�5A, B). These optimization steps enabled the preparation of isotopically labeled holoproteins of RcaE at amounts sufficient for resonance Raman and NMR analyses via upscaling of the *E. coli* culture. The cyanobacteriochromes AnPixJ and NpR6012g4 are members of the red/green subfamily that photoconverts between Pr and Pg with fully protonated PCB (Narikawa et al. 2008, Rockwell et al. 2015). For AnPixJ, there are spectral differences among the UV–Vis, Fourier transform-infrared, resonance Raman and NMR analyses of samples prepared via in vitro reconstitution and PCB-producing *E. coli* (Song et al. 2015). Song et al. (2015) suggested that the occurrence of deprotonation of PCB in the green-absorbing Pg state of AnPixJ is caused by the misfolding of the holoprotein prepared via in vitro reconstitution. By contrast, we did not observe any differences in the UV–Vis spectrum of the RcaE holoprotein prepared via in vitro reconstitution and PCB-producing *E. coli* (Fig.�5F, G), when the difference in buffer pH was taken into account. Therefore, our in vitro reconstitution will be useful for isotopic labeling of RcaE and other PCB-binding proteins for various spectroscopic analyses.

## Materials and Methods

### Strain and growth conditions

The motile substrain P of *Synechocystis* was used for PCB production (Yoshihara et al. 2000). *Synechocystis* cells were grown in 2 l of BG11 medium (Stanier et al. 1971) supplemented with 20 mM HEPES-NaOH, pH 7.8. The cells were cultured at 30�C under red LED illumination (655 nm peak at 0.6 mW intensity) with bubbling air containing 1% (v/v) CO_2_ with stirring. For isotopic labeling with ^15^N, 4.36 mM Na^15^NO_3_ (98%+, Cambridge Isotope Laboratories) was used for the nitrogen source and the BG11 medium was sterilized with an autoclave. The cells were grown under the same conditions as for the non-labeled BG11 medium. For dual labeling with ^13^C and ^15^N, 12.5 mM NaH^13^CO_3_ (99%, Cambridge Isotope Laboratories, Inc.) and 2.18 mM Na^15^NO_3_ were utilized as the carbon and nitrogen sources, respectively. HEPES-NaOH (20 mM, pH 7.8) was replaced with 100 mM CHES as reported previously (Strauss et al. 2005), and citric acid was omitted from the BG11 medium. The modified BG11 medium for ^13^C and ^15^N labeling was sterilized via filtration using the Stericup Quick Release-GP Sterile Vacuum Filtration System (Merck, USA). The cells were grown in 8 l of modified BG11 medium in a sealed bottle with stirring but without bubbling.

To optimize the concentrations of NaH^13^CO_3_ and Na^15^NO_3_ for ^13^C/^15^N labeling, we compared 16 combinations with different concentrations of non-labeled NaHCO_3_ and NaNO_3_: 100, 50, 25 and 12.5 mM for NaHCO_3_ and 17.4, 8.72, 4.36 and 2.18 mM for NaNO_3_. *Synechocystis* cells (1 ml) were transferred to 50 ml of modified BG11 medium in a 100-ml flask and cultured on an orbital shaker (150 rpm) at 30�C with red light LED illumination. Cell density was measured and chlorophyll *a* and phycocyanin concentrations were calculated at 24-h intervals as previously reported (Arnon et al. 1974) based on absorption data from a spectrophotometer (Model V-650; JASCO, Japan) equipped with a transmission integrating sphere (Model ISV-722; JASCO). These values are reported as the 3-day moving average to mitigate variations in measurement owing to the propensity of the *Synechocystis* P strain to aggregate.

### Extraction of PCB with PLE of Lina Blue and *Synechocystis* cells

PCB was extracted from Lina Blue G1 powder (DIC LifeTech, Japan) or *Synechocystis* cells (Fig.�1). Lina Blue contained 40% (w/w) phycocyanin purified from *Spirulina*, 55% (w/w) trehalose and 5% (w/w) sodium citrate according to the manufacturer’s data. First, 0.5 g Lina Blue was resuspended in 3 ml methanol or ethanol in a 15-ml tube, vortexed and centrifuged at 18,000 � *g* for 10 min at 20�C. This wash step was carried out six times with methanol and then two times with ethanol to remove the trehalose and sodium citrate. The washed pellets were mixed with 1.5 g Celite 545 (Kanto Chemical, Japan) in a mortar and transferred into a 40-ml vessel of an E-916 speed extractor (B�CHI Labortechnik AG, Switzerland) with 2.0 g and 40 g silica sand on the bottom and top, respectively (Fig.�1). PCB was extracted for 5 min in 99.5% (v/v) ethanol under 100-bar pressure at 100, 125, 150 or 200�C, with a 2-min elution by fluxing of nitrogen gas. The cycle of extraction and elution was carried out four times. For PCB extraction from *Synechocystis*, the cells were harvested by centrifugation at 14,000 � *g* for 10 min at 20�C, washed twice with 50 ml methanol and washed with 25 ml ethanol until the supernatant became colorless (Fig.�1). Then, ∼0.5 g (wet weight) of each blue cell pellet was mixed with 1.5 g Celite in a mortar and extracted with E-916. One extraction was performed for 2 min at 75�C (prewash) to remove the remaining non-covalently linked pigments (e.g. carotenoids) of *Synechocystis*. Then, PCB was extracted for 5 min in 99.5% (v/v) ethanol under 100 bars and 125�C with a 2-min elution by fluxing of nitrogen gas, and these steps were carried out three times. Absorption spectra of the PCB extracts were measured using the V-650 spectrophotometer. Uncleaved PCB and cleaved C3-*E* and C3-*Z* PCB have different structures and therefore have slightly different absorption peak maxima and molecular extinction coefficient values. In this study, extraction efficiency of PCB was calculated with the following equation with the heuristic assumption that uncleaved PCB and cleaved C3-*E* and C3-*Z* PCB have the same molecular extinction coefficient in their red-absorption peaks in 8 M urea pH 2.0. The PCB solution in ethanol was evaporated at 35�C under dim light and then stored at −80�C.

$$\text{Extraction}�\text{efficiency}�(\%)=(A_{694-1\text{st}}�V_{1\text{st}}+A_{694-2\text{nd}}�V_{2\text{nd}}+A_{694-3\text{rd}}�V_{3\text{rd}})/(A_{663-\text{pellet}}�V_{\text{pellet}})�100$$

*A*_694-1st_, *A*_694-2nd_ and A_694-3rd_: absorption measurements at 694 nm for the PCB extracts from the 1st, 2nd and 3rd extractions, respectively.

*A*_663-pellet_: absorption measurements at 663 nm for the Lina Blue before the extraction in 8 M urea pH 2.0.

*V*_1st_, *V*_2nd_ and *V*_3rd_: total volumes (ml) of the PCB extracts from the 1st, 2nd and 3rd extractions, respectively.

*V*_pellet_: total volume (ml) of the Lina Blue before the extraction in 8 M urea pH 2.0.

### LC–MS analysis

The PCB extracts were analyzed with LC–MS using the e2695 system (Waters, USA) equipped with a Model 2998 photodiode array detector, Model 2475 fluorescence detector and an Acquity QDa detector. The absorption spectra (300–800 nm) were monitored using the photodiode array. The fluorescence emission at 660 nm was monitored with an excitation at 350 nm using fluorescence detection. Mass detection by the QDa detector was performed using the positive scan mode at a probe temperature of 300�C, cone voltage 20 V and capillary voltage 1.2 kV. PCB extracts (10 �l each) in ethanol were separated on a X-Bridge C18 column (4.6 mm � 150 mm, 3.5 �m particle size, Waters) with a linear gradient from 40% (v/v) to 60% (v/v) acetonitrile in water containing 0.01% (v/v) formic acid for 15 min at a 1.0 ml/min flow rate with a column temperature of 35.0�C. For the analysis of the yellow-colored prewash fraction (Fig.�4A), the extracts were concentrated with a vacuum concentrator and passed through a 0.45-�m filter to remove yellow precipitation. The flow-through fraction was analyzed with an additional gradient from 60% (v/v) to 95% (v/v) acetonitrile for 10 min with a continuous flow of 95% (v/v) acetonitrile for 10 min (Supplementary Fig. S4). Chromatograms were analyzed using Empower 3 software (Waters).

### In vitro reconstitution of PCB with RcaE

The apoprotein of the PCB-binding GAF (cGMP phosphodiesterase/adenylyl cyclase/FhlA) domain of RcaE was expressed in the *E. coli* C41(DE) strain as described previously (Hirose et al. 2013). To optimize the expression conditions, *E. coli* cells were cultured at 37�C for 2 h in 1 l Luria–Bertani medium that was then supplemented with 0.01 or 1 mM isopropyl *β*-d-1-thiogalactopyranoside (IPTG) and cultured at 16 or 25�C for 16.5 h, or at 37�C for 2 h. Cells were harvested by centrifugation at 8,273 � *g* for 10 min and resuspended in 50 ml disruption buffer [20 mM HEPES-NaOH, pH 7.4, 100 mM NaCl, 1 mM dithiothreitol (DTT) and 0.1% (v/v) Tween 20]. Each suspension was disrupted with a French press (5501 Cells, Ohtake, Japan) for three times at 15,000 psi. Then, 2 �M of the PCB extracted from Lina Blue was added to an *E. coli* lysate, incubated for 10 min on ice and then centrifuged at 20,000 � *g* for 10 min at 4�C. The supernatant was illuminated by green LEDs emitting 515-nm light at 100 mW or by red LEDs emitting 620-nm light at 120 mW for 30 s. Difference absorption spectra were measured using the V-650 spectrophotometer with a transmission-integrating sphere. PCB extracts of Lina Blue from the 1st, 2nd and 3rd extractions at 125�C were mixed, concentrated by evaporator and used for in vitro reconstitution experiments. PCB concentration was calculated using a molar extinction coefficient of 37,900 M/cm at 680 nm for C3*-E* PCB in 36% (w/v) aqueous HCl/methanol (1:49, v/v) (Cole et al. 1967). *E. coli* cells grown at 16�C with IPTG induction (0.01 mM) in 1 l Luria–Bertani medium gave the highest yield and therefore were used in subsequent experiments.

To optimize the concentrations of detergents, reducing reagents and PCB, *E. coli* cells were disrupted in 50 ml of 20 mM HEPES-NaOH, pH 7.4, containing 100 mM NaCl. Then, 1-ml aliquots of *E. coli* lysate were supplemented with DTT (1 or 10 mM), Tween 20 [0.1 or 1% (v/v)] and/or Triton X-100 [0.1 or 1% (v/v)], followed by the addition of 2 �M PCB. To optimize the PCB concentration, 0.321, 0.642, 0.963, 1.28, 1.61, 1.93, 2.25, 2.57, 2.89 or 3.21 �M PCB was added to 1-ml aliquots of *E. coli* lysate in the presence of 1 mM DTT and 0.1% (v/v) Tween 20. Difference absorption spectra of the *E. coli* lysate after green and red illumination were measured as described above. To monitor the time course of covalent linkage formation of PCB, the apoprotein of RcaE in 1 ml *E. coli* lysate was reconstituted with 2 �M PCB at room temperature, and then 30-�l aliquots were sampled from 20 s to 15 min and denatured in sample buffer for SDS-PAGE [62.5 mM Tris-HCl, pH 6.8, 2% (w/v) SDS, 5% (v/v) β-mercaptoethanol, 5% (w/v) sucrose and 0.005% (w/v) bromophenol blue]. SDS-PAGE, CBB staining and detection of the covalently linked PCB in the presence of Zn^2+^ were performed as described previously (Sato et al. 2019). Fluorescence was detected with a Typhoon FLA 9400 imager (GE Healthcare, USA) with a 488-nm excitation and a 655–685-nm band-pass filter. The relative florescence intensity of each band was quantified using ImageQuant TL software (GE Healthcare).

For purification of the reconstituted RcaE holoprotein, *E. coli* cells were disrupted in 50 ml disruption buffer containing 2 �M PCB, incubated for 10 min on ice and centrifuged at 164,700 � *g* for 30 min at 4�C. The supernatant was subjected to Ni-affinity chromatography purification as described previously (Sato et al. 2019). The eluted colored fractions were collected, concentrated using centrifugal filters (Amicon Ultra-0.5 ml, 10 kDa; Merck) and further purified by gel-filtration chromatography using a HiPrep 16/60 Sephacryl S-300 HR column (GE Healthcare) in disruption buffer at 0.5 ml/min. The absorption spectra of the RcaE holoprotein prepared via in vitro reconstitution were compared with those prepared with PCB-producing *E. coli* in our previous study (Hirose et al. 2013). Protein concentration was measured using a Bradford Protein Assay kit (Takara Bio, Japan).

## Supplementary Data

Supplementary data are available at PCP online.

## Funding

A Grant-in-Aid for Scientific Research (C) (grant number 19K06707) to Y.H. from the Japan Society for the Promotion of Science (JSPS) and research grants to Y.H. from the Foundation for the promotion of Ion Engineering and Engineering and JGC-S Scholarship Foundation.

## Supplementary Material

pcaa164_Supplementary_Data
