# Supplementary figures and images for: Pressurized Liquid Extraction of a Phycocyanobilin Chromophore and Its Reconstitution with a Cyanobacteriochrome Photosensor for Efficient Isotopic Labeling
Source: Plant Cell Physiol. 2021 Jan 2;62(2):334–47. doi: 10.1093/pcp/pcaa164 (PMC8112840; doi:10.1093/pcp/pcaa164)

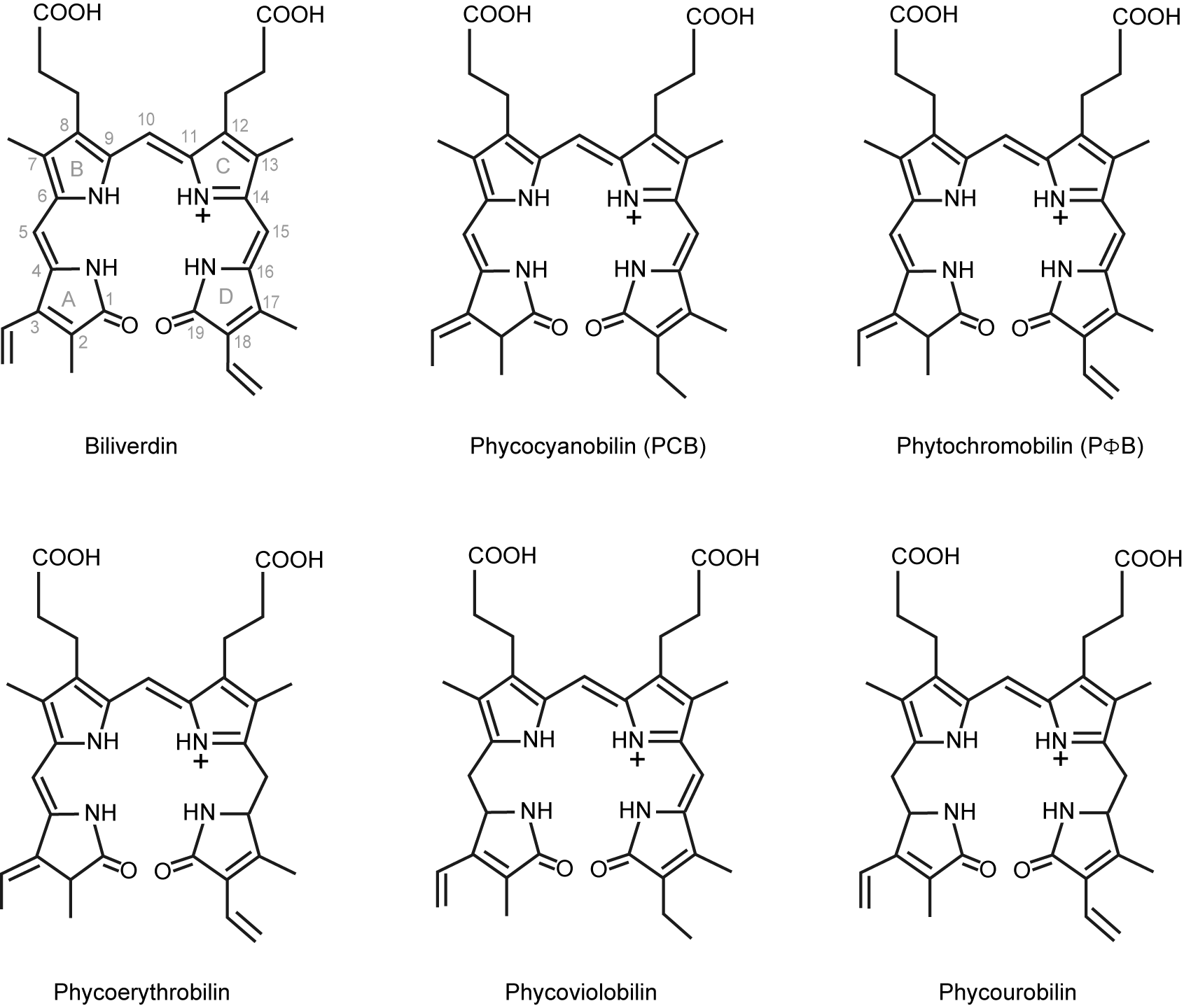

Supplement: pcaa164_Supplementary_Data [file pcaa164_supplementary_data.zip › pcp-2020-e-00422-File007.tif]

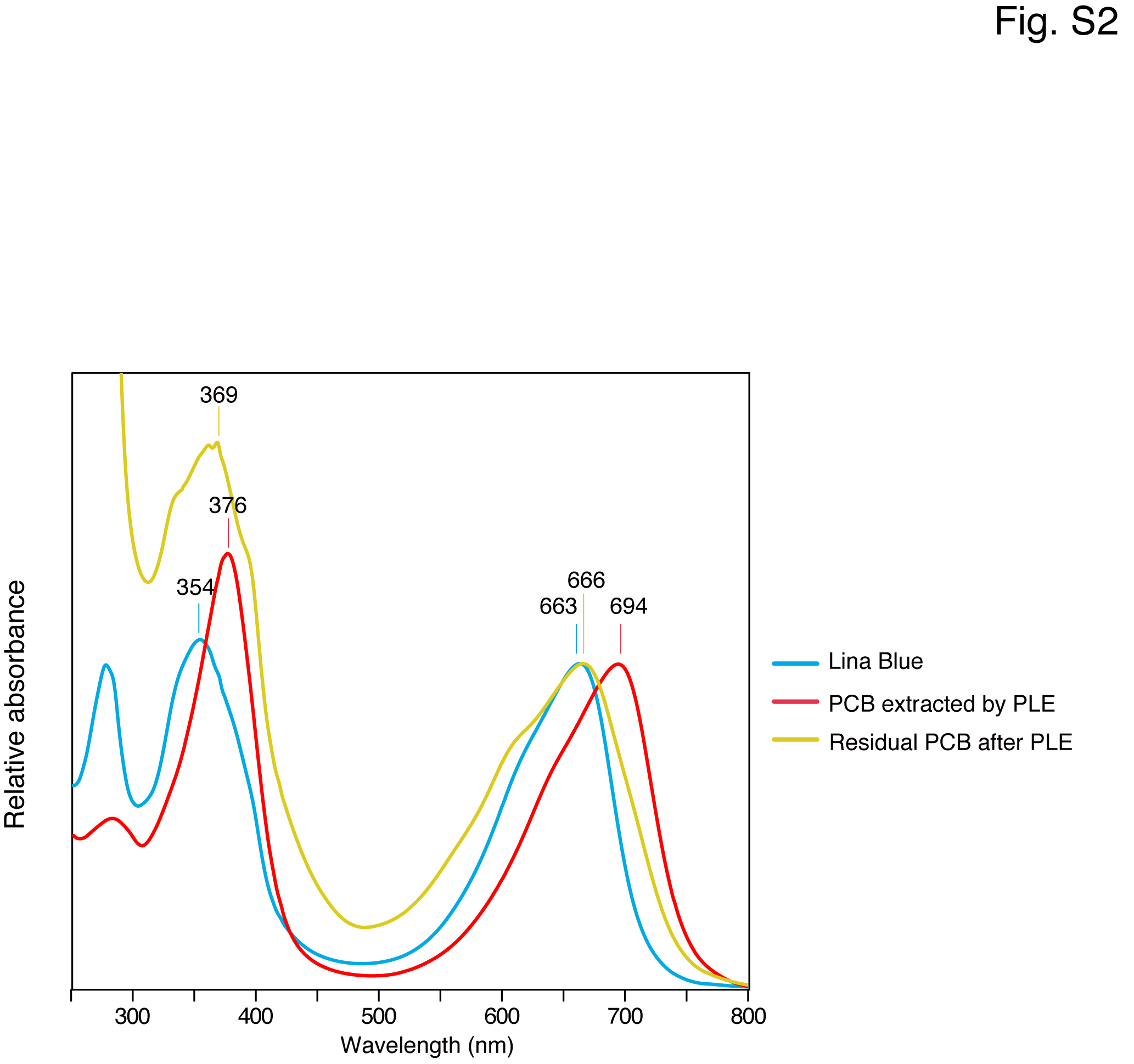

Supplement: pcaa164_Supplementary_Data [file pcaa164_supplementary_data.zip › pcp-2020-e-00422-File008.tif]

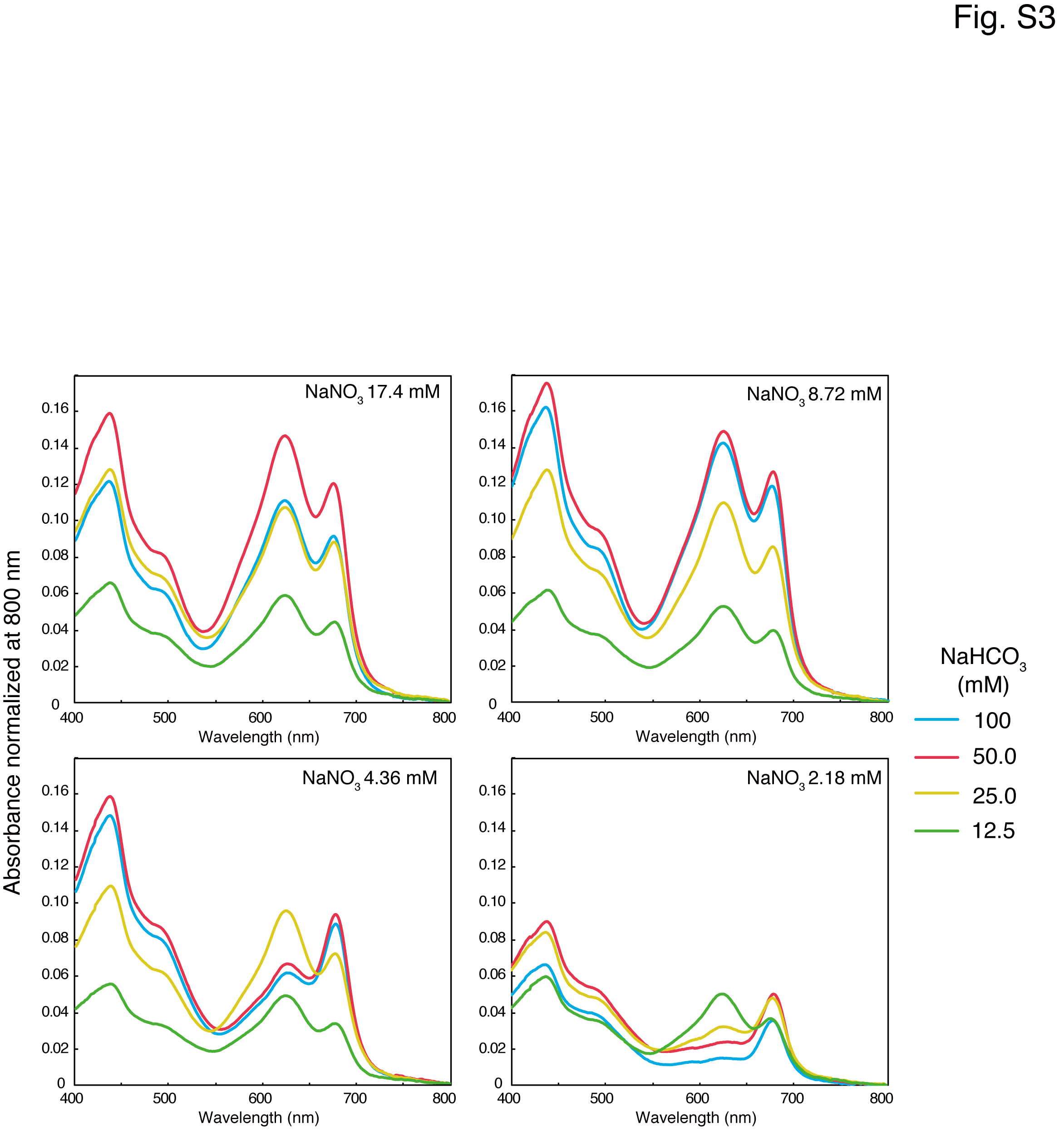

Supplement: pcaa164_Supplementary_Data [file pcaa164_supplementary_data.zip › pcp-2020-e-00422-File009.tif]

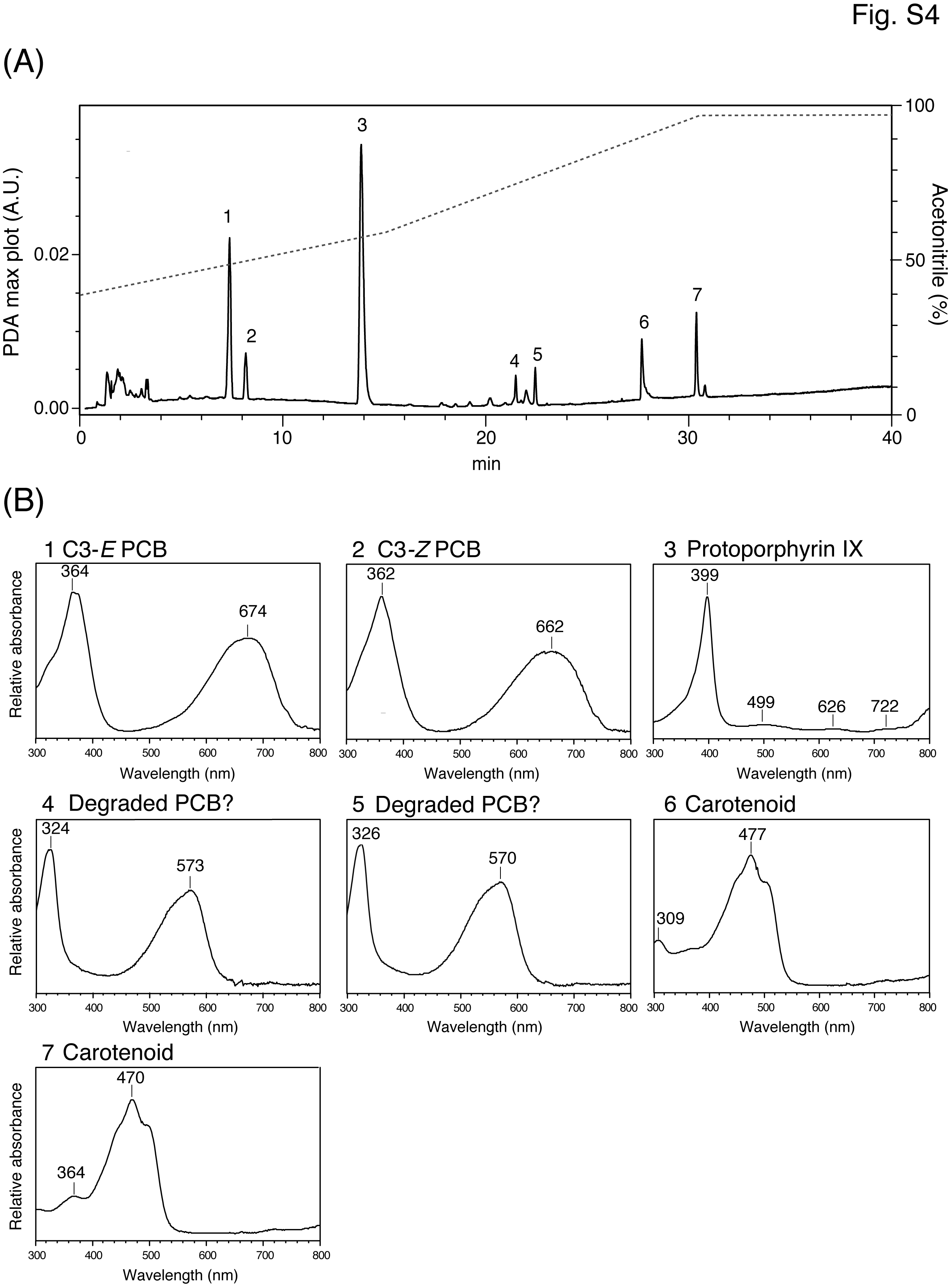

Supplement: pcaa164_Supplementary_Data [file pcaa164_supplementary_data.zip › pcp-2020-e-00422-File010.tif]

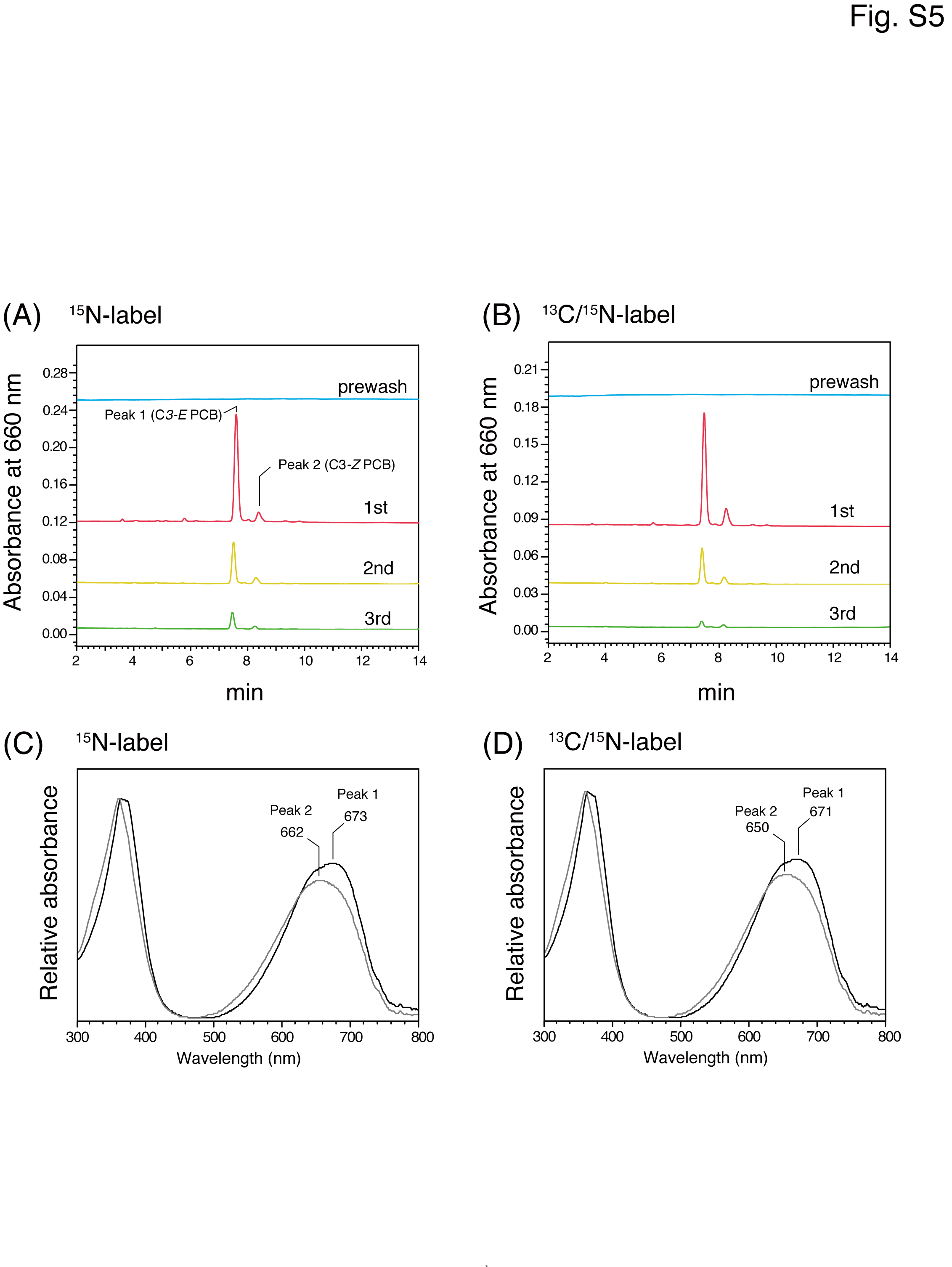

Supplement: pcaa164_Supplementary_Data [file pcaa164_supplementary_data.zip › pcp-2020-e-00422-File011.tif]

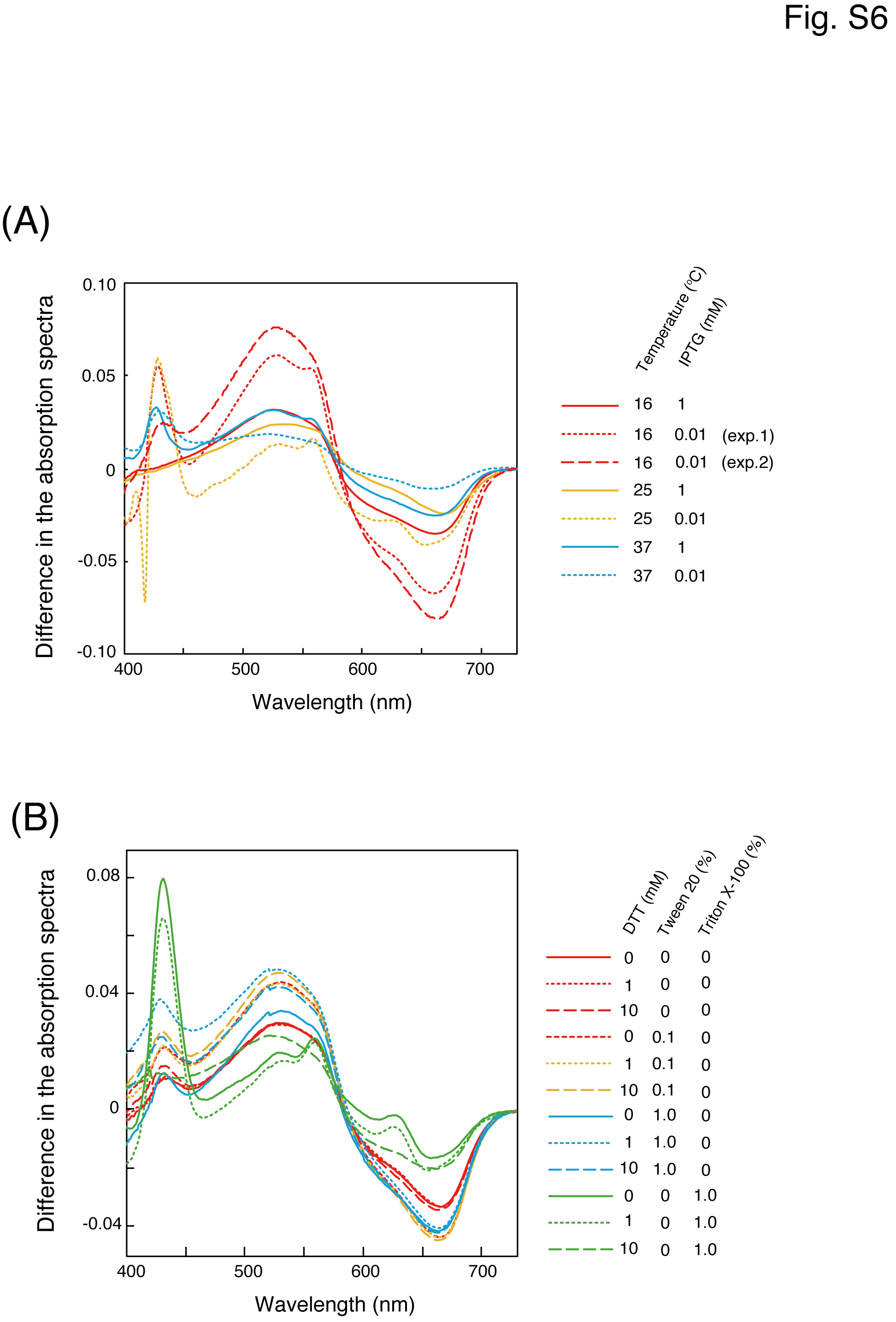

Supplement: pcaa164_Supplementary_Data [file pcaa164_supplementary_data.zip › pcp-2020-e-00422-File012.tif]

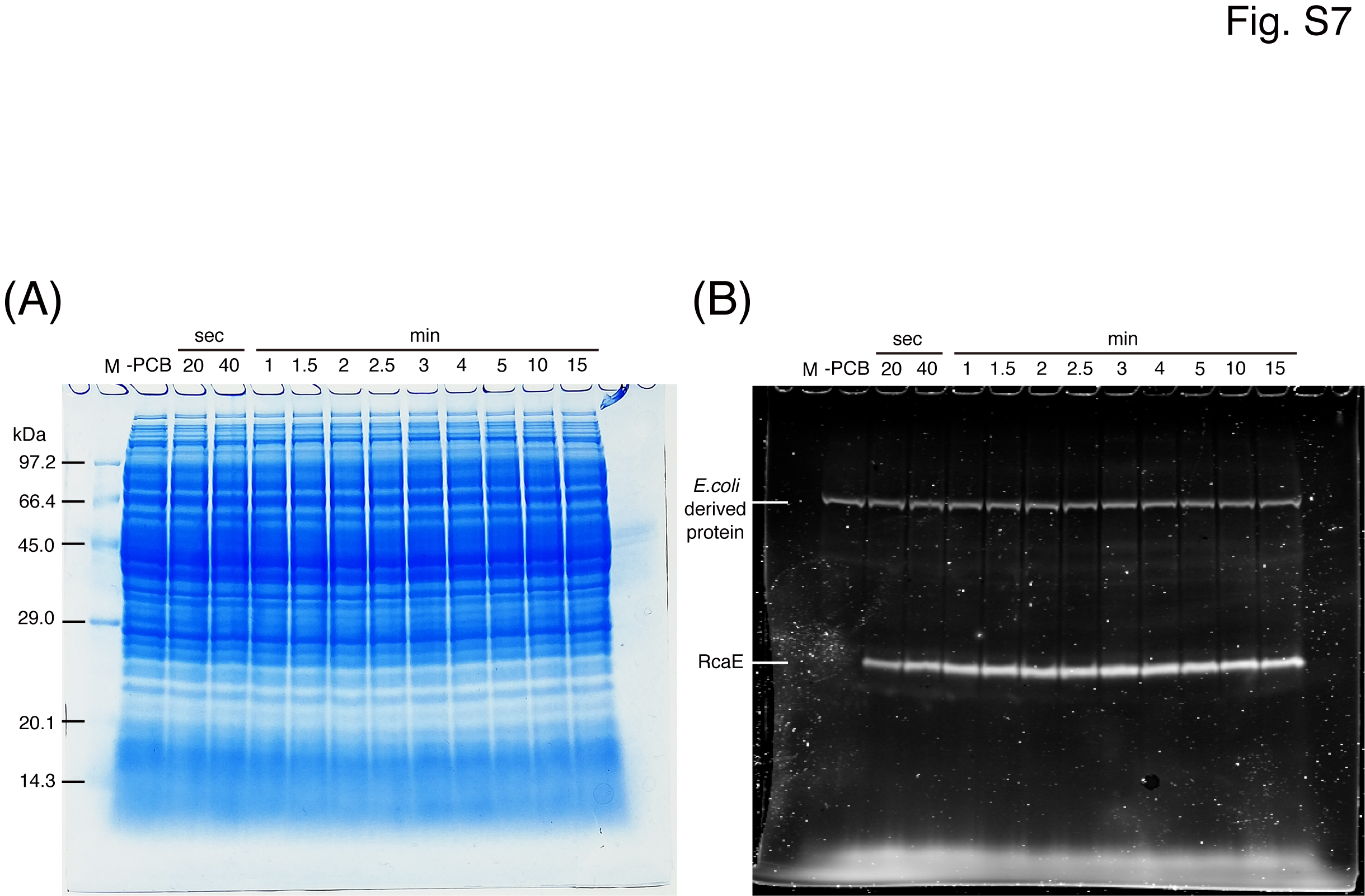

Supplement: pcaa164_Supplementary_Data [file pcaa164_supplementary_data.zip › pcp-2020-e-00422-File013.tif]
